# Burden and Predictors of Anaemia in Children 1 to 5 Years at the Ejura‐Sekyedumase Municipality in Ghana

**DOI:** 10.1155/anem/4726556

**Published:** 2026-06-26

**Authors:** Eugenia Sly-Moore, Samuel Blay Nguah, Haruna Mahama, Sampson Antwi

**Affiliations:** ^1^ Directorate of Child Health, Komfo Anokye Teaching Hospital, Kumasi, Ghana, kathhsp.org; ^2^ Department of Paediatrics, St Luke Hospital, Kasei, Ghana; ^3^ Department of Child Health, School of Medical Sciences, Kwame Nkrumah University of Science and Technology, Kumasi, Ghana, knust.edu.gh; ^4^ Department of Paediatrics, St. Theresa’s Hospital, Nandom, Ghana

**Keywords:** anaemia, burden, children, under-five

## Abstract

**Background:**

The burden and effects of anaemia remain high worldwide, with predictors varying by location, demographics, and socioeconomic factors. Knowing localised, specific predictors of anaemia helps develop targeted local interventions. We therefore set out to determine the prevalence and predictors of anaemia in a semiurban and rural community in Ghana.

**Methods:**

From June to August 2021, a community‐based cross‐sectional study using multilevel sampling was conducted in the Ejura‐Sekyedumase Municipality, Ghana. Four hundred and seventy‐six children, aged one to five, were recruited from four communities in the Kasei Subdistrict. Using a structured questionnaire, clinical, demographic, anthropometric, dietary and laboratory data, including blood haemoglobin, were obtained. The data were entered into EpiData and analysed with R statistical software. A multivariate ordinal logistic regression model was used to identify independent predictors of increasing anaemia severity.

**Results:**

The prevalence of anaemia was 76.3% (95% CI: 72.2–80.0), with mild, moderate and severe anaemia being 28.8% (95% CI: 24.7–33.1), 44.3% (95% CI: 39.8–48.9) and 3.2% (95% CI: 1.8–5.1), respectively. Two hundred and fifty (52.5%) children had malaria parasitaemia. Independent predictors of worsening anaemia severity were increasing age (aOR: 0.96, 95% CI: 0.95–0.98, *p* < 0.001), child eating from his/her bowl as opposed to communal eating (aOR: 0.47, 95% CI: 0.28–0.80, *p* = 0.005), stunting (aOR: 1.83, 95% CI: 1.17–2.88, *p* = 0.009), malaria parasitaemia (aOR = 3.62, 95% CI: 2.42–5.46, *p* < 0.001) and mothers’ religion (aOR: 2.44, 95% CI: 1.62–3.68, < 0.001).

**Conclusions:**

The Ejura‐Sekyedumase Municipality has a high burden of anaemia and malaria parasitaemia in children between one and five years old. Malaria parasitaemia, communal eating, younger age and the mother’s religion were significantly associated with worsening anaemia. It is recommended that intermittent antimalarial treatment, routine administration of iron and trace elements, and counselling at well‐baby and antenatal clinics on specific predisposing dietary habits be instituted for children under five years of age.

## 1. Background

Anaemia is a clinical condition characterised by a decrease in red blood cell count or haemoglobin content. Quantitatively, anaemia is defined as red blood cells, haemoglobin, or haematocrit less than the estimated age and sex ranges [1]. According to the World Health Organisation, using the cutoff of haemoglobin less than 11 g/dL, 40% of children between six months and five years are anaemic worldwide [2]. In Africa, the prevalence of anaemia in children under five is 59.9%, ranging from 23.7% in Rwanda to 87.9% in Burkina Faso [3]. The 2022 Demographic and Health Survey in Ghana estimated that the prevalence of anaemia in children under five years old was 49% in 2022, down from 79% in 2002 [4].

Regardless of the aetiology and contributing factors, the adverse consequences of anaemia in preschool children are well established. Anaemic children often experience dizziness and fatigue, reduced cognitive function, and slower growth rates [5]. Additionally, anaemia may lead to other detrimental effects on children aged 1–5 years, such as increased susceptibility to infections, impaired physical development and compromised immune function [6].

Considering the high prevalence of malnutrition in the West African subregion [7], it is unsurprising that iron deficiency, anaemia and malnutrition often coexist. Anaemia has also been associated with almost every aspect of a child’s diet, from exclusive breastfeeding and the introduction of complementary feeds to the initiation of an adult diet. Furthermore, anaemia in children under five is strongly associated with other childhood, parental and economic factors such as the child’s age, birth order, presence of other intercurrent illnesses and sex [8]. Maternal education and age are the most prominent parental factors [3]. Other factors include the community in which the child lives and recent medical conditions such as diarrhoea, fever, respiratory infections, parasites and helminth infestations [9].

Most residents in Ejura‐Sekyedumase are crop farmers who grow protein‐ and iron‐rich foods such as beans and groundnuts. It is unclear whether these foods are used in their children’s diets or are primarily sold to meet other family needs. Furthermore, it is uncertain whether medical conditions like malaria, intestinal worms and malnutrition due to poor feeding contribute to anaemia in this semi‐urban community.

In Ghana, anaemia is highly prevalent among children under five, with notable regional disparities. Data show the Ashanti Region has rates comparable to or higher than national levels [4]. However, district‐level data, especially from rural areas like Ejura‐Sekyedumase, are limited. Most studies rely on national surveys or hospital data, which may not reflect community‐level risks. This gap in localised evidence highlights the need for further research.

Finally, although surveys in Ghana link anaemia to many factors in childhood, community data rarely explain why it persists in rural districts like Ejura‐Sekyedumase. Few studies concurrently examine the relationship between anaemia and malaria, dietary practices such as communal eating, and sociodemographic factors, including maternal education and religion, in a typical Ghanaian suburban and rural community. This study, therefore, aims to determine the burden of anaemia and its contributing factors among children aged 1–5 years in selected communities in the Ejura‐Sekyedumase Municipality, Ashanti Region, Ghana.

## 2. Methods

### 2.1. Study Settings

A community‐based cross‐sectional study was conducted in four selected communities within the Kasei Subdistrict of the Ejura‐Sekyedumase Municipality. The Kasei Subdistrict was selected by simple random sampling from the seven subdistricts in the municipality to minimise selection bias and improve representativeness. The prevailing climatic conditions and topography of the area are favourable for the cultivation of food crops, especially cereals. Most people living within the municipality are farmers, with corn, millet, sorghum, rice, beans, groundnuts, yams, plantain, cassava, and green leafy vegetables being the most common crops cultivated. The municipality has seven subdistricts, 125 communities, 12 health facilities, 116 outreach sites, 43 Community‐based Health Planning and Services (CHPS) zones, and an estimated population of 137,672, of which approximately 23,650 are under 5 years old [10, 11].

### 2.2. Study Design and Participants

A community‐based cross‐sectional study was used in 4 selected communities in the Kasei Subdistrict, one of the seven subdistricts in the municipality, from June to August 2021. The study population consisted of all children aged 1 to 5 years living in the subdistrict. The total number of these eligible children was 2,703, according to the Ejura‐Sekyedumase Municipal Health Profile 2021 [12]. All children who lived in the selected communities of Kasei (Masuo, Sunkwae, Kasei and Dromankuma) and whose parents consented to participate formed the study population. Among the children excluded from the study were those who had not lived in the selected communities for at least six months or were suffering from an acute illness at the time of the survey.

### 2.3. Sample Size Determination

Since recent regional or district anaemia prevalence data for children in the Kasei Subdistrict aged one to five were unavailable at the design of the study, the national prevalence estimate from the Ghana Demographic and Health Survey of 70% [13] was used to determine the sample size. Using a precision of 0.06 from the estimate, a 95% confidence level, and a design effect of 2, the minimum sample size for the study was 448. In determining our sample size, we employed the formula for a quantitative cross‐sectional study as published by Charan and Biswas [14]. We then applied the design effect as a multiplier to account for clustering.

### 2.4. Sampling Technique

Multistage sampling was done. First, using simple random sampling, a subdistrict (Kasei) was selected from the seven in the Ejura‐Sekyedumase district. Twenty‐five communities within the previously sampled Kasei Subdistrict were grouped into 8 zones. The second sampling stage involved selecting four out of the eight zones by simple random sampling. This led to the selection of the Masuo, Sunkwae, Kasei and Dromankuma communities. The estimated population of children aged 1–5 years across these four selected zones was 1217. This was based on the 2010 population census [11]. The sample size was distributed across the four communities in proportion to their child populations aged 1 to 5 years. Thus, Dromankuma, Kasei, Masuo and Sunkwae, with estimated populations of 569, 325, 161 and 160, were allocated 211, 120, 59 and 58, respectively. The next sampling stage was selecting households. This third sampling stage involved a systematic random sampling of households from which participants were recruited. Households in each community were selected through systematic random sampling. Due to the lack of an official household list, a field‐based method was employed. The research team chose a central landmark, such as a school, health post, or market, in each community. A random direction was determined, and all households along that line were numbered sequentially. Local health volunteers helped estimate the total number of households. The sampling interval (k) was calculated by dividing this estimate by the desired number of participants from the community. A random starting point within the first interval was selected, and every kth household was visited until the target sample size was reached. If a household did not have an eligible child aged one to five years, the next household was visited, maintaining the systematic pattern. Within a particular community, one child was to be recruited from the selected household. If there were no eligible children in a household, the household was skipped, but the sampling interval was preserved. If there were more than one eligible child in a household, a simple ballot was used to select one child.

### 2.5. Study Procedure and Methods of Data Collection

Data were collected using a pretested structured questionnaire. Before the start of the study, the questionnaire was generated using the data from previous similar publications and taking into consideration the peculiar demographics of the communities to be sampled. It was pretested on 20 children, and adjustments were made. Before initiating the study, all study personnel were trained in its administration and other procedures to be used during the study. Training was only finalised after the personnel passed predefined knowledge and competency tests. The questionnaire captured the children’s sociodemographic characteristics, feeding habits, clinical history, anthropometric measurements, and information about their parents or guardians. Four trained nurses and two certified laboratory technicians were recruited and trained to collect specimens and data.

Before the team arrived in the various communities, the principal investigator informed the chiefs and elders and sought their permission. The communities were then notified of the study team’s impending arrival and purpose. The visit to the various communities was planned to avoid coinciding with specific farming and marketing days. This maximised the chance of meeting the child and parents at home. Upon arrival at the selected households, the study team introduced themselves and their purpose. After the parents agreed to participate, the contents of the informed consent form were explained in their preferred language, after which they either signed or thumb‐printed. A team member then administered the structured questionnaire to the carer. Each participant was given an ethylenediaminetetraacetic acid (EDTA)‐coated bottle and a wide‐cap container for collecting blood and stool samples, respectively.

### 2.6. Anthropometry Measurements

The children were weighed barefoot on a calibrated Rossmax digital weighing scale (serial number: WF260). Measurements were taken in kilograms (kg) to the nearest 0.1 kg. The length of children under two years was measured with an infantometer by placing their heads at the zero mark and laying them straight on their backs with arms and legs together. The flat distal base/board was then drawn to the soles of their feet to mark their length. The heights of children older than 2 years were measured using the same stadiometer from Narag Medical Limited with serial number SLH1‐21. The child stood barefoot on the device’s base plate, with a straight back against the measuring rod, hands at their sides, looking straight ahead. The head plate was then drawn to the vertex of the head to read the corresponding height in centimetres (cm) to the nearest 0.1 cm. The Ghana Standards Authority calibrated these measuring instruments before the start of the study. The child’s nutritional status was estimated using the WHO growth standards for children, interpreted as being underweight, wasted, or stunted (i.e., weight‐for‐age, weight‐for‐height and height‐for‐age Z‐score < ‐2 SD), respectively [15].

### 2.7. Haematological Measurements

A tourniquet was used to tie the arm of the child while the nurse cleaned the dorsum of the hand with cotton soaked in 70% methylated spirit. The hand of the child was held securely in a fist position, and approximately 1.5 mL of venous blood was collected from each participant into EDTA‐coated bottles. The sample, with the requisite identification number, was placed in a clear, waterproof ziplock bag and placed in an ice chest in readiness for transport to the laboratory that same day. Upon arrival, the samples were stored on a rack in the refrigerator. The haemoglobin, mean corpuscular volume and other haematological parameters were determined within 24 h using an automated haematology analyser, Mindray BC 3000plus (HGTU with serial number 2934674501). Used slides and consumables were disposed of in a puncture‐resistant sharps container. Also, infectious waste was autoclaved before being finally disposed of. Anaemia was defined as haemoglobin levels less than 10.5 and 11.0 g/dL for children between 12 and 23 months and 24 and 59 months, respectively. For children less than 24 months, mild, moderate and severe anaemia were defined as haemoglobin levels between 9.5 and 10.4, 9.4 and 7.0 g/dL, and less than 7.0 g/dL, respectively. For those 24 months and older, mild, moderate, and severe anaemia were defined as 10.0 to 10.9 g/dL, 7.0 to 9.9 g/dL and less than 7.0 g/dL. This categorisation was based on the 2024 WHO guidelines for determining anaemia [16].

### 2.8. Stool Sampling and Examination

A prelabelled sterile, disposable, wide‐cap, spatula‐fitted container was used for stool collection. The containers had well‐fitting, leak‐proof lids and were without disinfectant or detergent residue. About 1.5 to 2 mg of stool samples were collected and preserved in an insulated container with ice packs. If the research team was unable to obtain stool during the current visit, mothers were advised to collect the samples the next morning. A team member returned to the community the following morning to collect all outstanding stool samples. All samples were therefore less than four hours old. Stool samples were handled and examined by the MSD Manual [17]. They were kept in an insulated container with ice packs to preserve them before being transported to the laboratory for analysis. For each stool sample, saline and iodine wet mount preparations were made and examined under 10x and 40x objectives by a certified laboratory technician who was part of the study team. Each sample was examined once to identify intestinal parasites. For quality assurance, one out of every 10 samples examined was randomly selected by a senior laboratory technologist and rechecked. After examination, all stool samples and related disposable materials were classified as biomedical waste and properly disposed of in accordance with standard infection prevention protocols. Specifically, the stool residues were first autoclaved and then disposed of in a designated biomedical waste pit at the laboratory facility under the supervision of the laboratory technologist.

### 2.9. Malaria Diagnosis

Malaria parasite testing was conducted in the laboratory using a rapid diagnostic test (RDT), an SD Bioline Malaria Ag P.f test kit, and microscopy. For the RDT, a drop of blood from the EDTA bottle was dropped into the sample well on the cassette, and two drops of buffer were added to the buffer well. The result was read after 20 min as positive or negative per the manufacturer’s instructions.

Slide processing for malaria parasitaemia was performed according to the WHO manual on parasite counting [18]. To detect malaria parasites by microscopy, thin and thick blood films were prepared. Three and 6 μL of blood were used to prepare the thin and thick blood films within 4 h after bloodletting. The blood films were labelled and air‐dried horizontally. The thin film was fixed with methanol. A 10% Giemsa (1:9) stock reagent diluted with phosphate buffer (pH 7.2) was used for staining for 10 min. The slides were then placed on a slide drying rack to dry thoroughly at room temperature before being examined under the light microscope. Two independent laboratory scientists then examined the slides under x100 immersion oil to detect malaria parasites. In cases of discordant results, the slide was reviewed by a senior laboratory technologist, and a consensus result was recorded.

For data analysis, malaria parasites were considered present if the RDT was positive or if malaria parasites were seen on the blood film.

### 2.10. Clinical Symptoms Evaluation

As part of the interviews, clinical symptoms such as fever, rhinorrhoea, cough and bloody stool were ascertained from the legal guardian or parent. They were considered present if the regular guardian or parent reported that it occurred within the last three months.

### 2.11. Data Management and Analysis

All data collected were manually entered into a database designed using EpiData Version 4.0.3 [19]. The data were regularly checked and cleaned of abnormal entries. Upon completion of data entry, the data were transferred to R statistical software Version 4.20 [20] for analysis. The prevalence of anaemia and its various levels were presented as proportions with exact binomial confidence intervals. Less than 1% of the data were missing and were imputed using multiple imputation by chained equations. Categorical variables, such as marital status, were analysed and presented as percentages. In contrast, continuous variables, such as age, were analysed and presented as means with their standard deviations or as medians with their interquartile ranges. All possible predictors were summarised, stratified by anaemia severity and presented in a tabular format. The association between these factors and the severity of anaemia was determined using the chi‐square test for trend for categorical variables and univariate ordinal regression for the continuous variables. The independent variables statistically significantly associated with anaemia severity were then put into a single multivariate ordinal regression to determine variables independently associated with anaemia severity. These associations were expressed as odds ratios with their respective confidence intervals. A two‐sided *p* value less than 0.05 was considered a statistically significant relationship for all analyses. The model assumptions, including collinearity, were checked for all regressions performed. At the multivariate stage, paternal religion was excluded due to its strong correlation with mothers’ religion.

## 3. Results

Overall, 476 study participants were recruited and analysed. Males formed the majority (252, 52.9%). The mean (SD) age of the study participants was 36.7 (14.9) months (Table 1). 94 (19.7%), 135 (28.4%), and 48 (10.1%) were underweight, stunted, or wasted, respectively. Malaria parasitaemia was seen in 250 (52.5%). Fever and a visit to a health facility within the past three months were seen in 316 (66.4%) and 156 (33.8%), respectively. Three children had intestinal helminths isolated from their stool samples, which were not associated with anaemia.

**TABLE 1:** Distribution of clinical, demographic and dietary characteristics of study participants and anaemia categories.

Characteristic	Anaemia categories				Overall, *N* = 476^1^	*p* value
	Normal, *N* = 113^1^	Mild, *N* = 137^1^	Moderate, *N* = 211^1^	Severe, *N* = 15^1^		
Community						
Kasei	37 (32.7)	40 (29.2)	43 (20.4)	4 (26.7)	124 (26.1)	
Dromankuma	64 (56.6)	55 (40.1)	103 (48.8)	7 (46.7)	229 (48.1)	0.174
Asuogya	4 (3.5)	25 (18.2)	32 (15.2)	0 (0.0)	61 (12.8)	**0.014**
Sunkwae	8 (7.1)	17 (12.4)	33 (15.6)	4 (26.7)	62 (13.0)	**0.002**
Sex, male	57 (50.4)	74 (54.0)	112 (53.1)	9 (60.0)	252 (52.9)	0.613
Child’s family size	4.9 (1.8)	5.2 (1.9)	5.3 (2.0)	5.1 (1.8)	5.2 (1.9)	0.119
Number of siblings	2.2 (1.9)	2.5 (1.8)	2.8 (2.1)	2.6 (1.6)	2.5 (2.0)	**0.013**
Age in months	36.5 (16.5)	39.6 (14.7)	35.2 (13.9)	31.8 (14.4)	36.7 (14.9)	**0.049**
Underweight	20 (17.7)	26 (19.0)	41 (19.4)	7 (46.7)	94 (19.7)	0.227
Low BMI	11 (9.7)	12 (8.8)	20 (9.5)	2 (13.3)	45 (9.5)	0.866
Stunting	19 (16.8)	40 (29.2)	67 (31.8)	9 (60.0)	135 (28.4)	**< 0.001**
Low MUAC	9 (8.0)	13 (9.5)	16 (7.6)	3 (20.0)	41 (8.6)	0.795
Wasting	14 (12.4)	12 (8.8)	19 (9.0)	3 (20.0)	48 (10.1)	0.719
Malaria parasite present	28 (24.8)	55 (40.1)	130 (61.6)	13 (86.7)	226 (47.5)	**< 0.001**
Dewormer given	59 (52.2)	70 (51.1)	103 (48.8)	5 (33.3)	237 (49.8)	0.315
Currently breastfeeding	27 (23.9)	21 (15.3)	42 (19.9)	4 (26.7)	94 (19.7)	0.833
Exclusive breastfeeding	75 (66.4)	93 (67.9)	153 (72.5)	12 (80.0)	333 (70.0)	0.146
Duration of exclusive breastfeeding	4.2 (2.3)	4.2 (2.2)	4.3 (2.2)	4.9 (2.1)	4.3 (2.2)	0.355
Age water given (months)	4.2 (2.3)	4.3 (2.1)	4.4 (2.1)	4.9 (2.1)	4.3 (2.2)	0.266
Age‐complimentary feed given (months)	5.7 (2.0)	5.8 (1.8)	5.9 (1.7)	6.2 (1.9)	5.9 (1.8)	0.249
Eats from own bowl	99 (88)	121 (88)	170 (81)	10 (67)	400 (84)	**0.013**
Meals eaten (day)	3.4 (0.6)	3.5 (0.8)	3.3 (0.5)	3.0 (0.6)	3.4 (0.6)	0.419
Meals eaten (night)	1.3 (1.7)	1.2 (1.5)	1.4 (1.4)	2.2 (1.9)	1.3 (1.5)	0.263
Eggs (days/week)	2.8 (2.3)	2.5 (2.3)	2.5 (2.3)	2.8 (2.4)	2.6 (2.3)	0.349
Milk (days/week)	3.0 (2.9)	2.6 (2.6)	2.5 (2.8)	2.5 (2.6)	2.7 (2.7)	0.105
Fruits (days/week)	2.9 (2.3)	3.1 (2.3)	2.4 (2.1)	3.2 (2.6)	2.7 (2.2)	0.073
Vegetables (days/week)	5.6 (2.0)	5.9 (1.8)	5.9 (1.8)	5.4 (2.1)	5.8 (1.9)	0.362
Fish (days/week)	6.0 (2.0)	5.8 (2.0)	6.0 (1.8)	5.5 (2.0)	5.9 (1.9)	0.728
Rhinorrhoea	62 (54.9)	78 (56.9)	113 (53.6)	6 (40.0)	259 (54.4)	0.474
Diarrhoea	58 (51.3)	70 (51.1)	107 (50.7)	10 (66.7)	245 (51.5)	0.778
Bloody stool	5 (4.4)	8 (5.8)	5 (2.4)	0 (0.0)	18 (3.8)	0.170
Blood in urine	0 (0.0)	1 (0.7)	1 (0.5)	0 (0.0)	2 (0.4)	0.761
Fever	66 (58.4)	91 (66.4)	148 (70.1)	11 (73.3)	316 (66.4)	**0.034**
Cough	49 (43.4)	68 (49.6)	101 (47.9)	8 (53.3)	226 (47.5)	0.486
Hospital visit	38 (33.6)	46 (33.8)	69 (32.7)	3 (20.0)	156 (32.8)	0.588
Hospital admission	23 (20.4)	24 (17.5)	40 (19.0)	2 (13.3)	89 (18.7)	0.735

*Note:* The bold values indicate statistically significant *p*‐values.

^1^ *n* (%); mean (SD).

### 3.1. Prevalence of Anaemia

Anaemia was observed in 363 (76.3%, 95% CI: 72.2–80.0) of the study participants. 137 (28.8%, 95% CI: 24.7–33.1), 211 (44.3%, 95% CI: 39.8–48.9), and 15 (3.2%, 95% CI: 1.8–5.1) had mild, moderate and severe anaemia, respectively.

### 3.2. Crude Predictors of Anaemia

Crudely, the community in which the patient lived was associated with the severity of anaemia. Increasing severity of anaemia was associated with an increasing number of siblings, younger age, stunting, malaria parasitaemia on a blood film, having a febrile episode within the past three months and communal eating. Parental factors associated with increasing severity of anaemia were mothers without formal education, either parent belonging to the Islamic faith, and the father being single Table 2.

**TABLE 2:** Distribution of parental characteristics of study participants and anaemia categories.

Characteristic	Anaemia categories				Overall, *N* = 476^1^	*p* value
	Normal, *N* = 113^1^	Mild, *N* = 137^1^	Moderate, *N* = 211^1^	Severe, *N* = 15^1^		
Mother’s age	27.4 (5.5)	28.3 (6.9)	27.0 (6.3)	27.6 (4.2)	27.5 (6.2)	0.504
Mother (single)	6 (5.3)	17 (12.4)	10 (4.7)	3 (20.0)	36 (7.6)	0.811
Mother’s education	79 (69.9)	83 (60.6)	113 (53.6)	6 (40.0)	281 (59.0)	**0.002**
Mother′s occupation: farmer	62 (54.9)	99 (72.3)	143 (67.8)	10 (66.7)	314 (66.0)	0.085
Mother’s religion						
Christian	65 (57.5)	70 (51.1)	78 (37.0)	5 (33.3)	218 (45.8)	
Islam	47 (41.6)	58 (42.3)	114 (54.0)	9 (60.0)	228 (47.9)	**< 0.001**
Others	1 (0.9)	9 (6.6)	19 (9.0)	1 (6.7)	30 (6.3)	**0.001**
Father’s age (years)	34 (7.9)	37 (10.3)	35 (7.9)	30 (5.4)	35 (8.1)	0.794
Father (single)	9 (8.0)	14 (10.2)	5 (2.4)	2 (13.3)	30 (6.3)	**0.050**
Father’s education	73 (64.6)	76 (55.5)	113 (53.6)	9 (60.0)	271 (56.9)	0.116
Father′s occupation: farmer	74 (65.5)	105 (76.6)	167 (79.1)	9 (60.0)	355 (74.6)	0.052
Father’s religion						
Christian	42 (37.2)	45 (32.8)	55 (26.1)	2 (13.3)	144 (30.3)	
Islam	54 (47.8)	64 (46.7)	118 (55.9)	9 (60.0)	245 (51.5)	**0.016**
Others	17 (15.0)	28 (20.4)	38 (18.0)	4 (26.7)	87 (18.3)	0.081

*Note:* The bold values indicate statistically significant *p*‐values.

^1^ *n* (%); mean (SD).

### 3.3. Independent Predictors of Anaemia Severity

After adjusting for all significant predictors, increasing child age was associated with significantly less anaemia (aOR: 0.81, 95% CI: 0.67–0.98, *p* < 0.03) Table 3. Children who ate alone from their bowl were significantly less likely to have anaemia when compared to those who practised communal eating (aOR: 0.49, 95% CI: 0.30–0.82, *p* < 0.01). Nutritionally, stunted children had significantly higher odds of anaemia than those who were not stunted (aOR: 1.70, 95% CI: 1.14–2.55, *p* = 0.01). Malaria parasitaemia was significantly associated with anaemia severity (aOR: 3.82, 95% CI: 2.57–5.72, *p* < 0.01). Finally, a mother belonging to the Islamic faith was associated with higher odds of anaemia compared to Christians (aOR: 2.44, 95% CI: 1.62–3.68).

**TABLE 3:** Univariate and multivariate association between significant characteristics and anaemia severity.

Characteristic	Univariate			Multivariate		
	COR	95% CI	*p* value	AOR	95% CI	*p* value
Community						
Kasei	1.00	—		—	—	
Dromankuma	1.32	0.88–1.99	0.174	0.85	0.54–1.33	0.474
Asuogya	1.99	1.15–3.47	**0.014**	1.23	0.67–2.28	0.505
Sunkwae	2.51	1.41–4.52	**0.002**	1.12	0.57–2.20	0.748
Age in months	0.86	0.73–1.02	0.080	0.81	0.67–0.98	**0.027**
Number of siblings	1.12	1.02–1.22	**0.013**	1.06	0.97–1.17	0.190
Eats from their own bowl	0.54	0.33–0.87	**0.013**	0.49	0.30–0.82	**0.006**
Stunting	1.90	1.31–2.78	**< 0.001**	1.70	1.14–2.55	**0.010**
Fever	1.47	1.03–2.09	**0.034**	1.22	0.84–1.78	0.294
Malaria parasite present	3.61	2.55–5.16	**< 0.001**	3.82	2.57–5.72	**< 0.001**
Mother’s education	0.58	0.41–0.81	**0.002**	0.88	0.60–1.29	0.508
Mother’s religion						
Christian	1.00	—		—	—	
Islam	1.82	1.29–2.58	**< 0.001**	2.44	1.62–3.68	**< 0.001**
Others	3.29	1.62–6.92	**0.001**	2.09	0.94–4.78	0.076
Father’s religion						
Christian	1.00	—				
Islam	1.60	1.09–2.34	**0.016**			
Others	1.55	0.95–2.53	0.081			
Father (single)	0.52	0.27–1.00	**0.050**	0.83	0.40–1.72	0.618

*Note:* The bold values indicate statistically significant *p*‐values.

Abbreviations: AOR, adjusted odds ratio; CI, confidence interval; COR, crude odds ratio.

## 4. Discussion

In our study, we set out to determine the prevalence and predictors of anaemia among children aged one to five in a suburban and rural community setting in Ghana. We had a relatively high prevalence of anaemia, 76.3%, as against the 2022 national prevalence of 49%. The independent predictors of anaemia in our population were younger children, engaging in communal eating rather than eating from individualised bowls, stunting in the child, malaria parasitaemia, and mothers belonging to the Islamic faith.

### 4.1. Prevalence of Anaemia

Several studies, both recent and past, have identified a high prevalence of anaemia in children under five, especially in developing countries such as Ghana. The findings from our study were not different. The four Demographic and Health Surveys conducted in Ghana in 2003, 2008, 2014 and 2022 have shown a decreasing trend in prevalence, with the latest survey reporting a prevalence of 49% [4, 13, 21]. These reports also note that the prevalence of anaemia varied significantly by region and locality; specifically, urban children had a lower prevalence than rural dwellers. Our study revealed a high prevalence of 76.3%, significantly deviating from the national average. Although most of our study population came from rural and suburban communities, this alone may not fully explain the finding. Other biological factors, such as the high parasitaemia observed in over half the children [22], stunting, seen in a significant number of children, dietary practices like communal eating, lack of maternal formal education, and religious affiliation, could all be contributing factors. Together, these findings indicate that the high prevalence is likely the result of a combination of nutritional, infectious and socio‐demographic influences, rather than geography alone.

In our study, and consistent with previous studies, moderate anaemia was the most prevalent of the three anaemia severities. For instance, in two studies from Ghana [23] and Tanzania [24], the prevalence of moderate anaemia was the most common at 48% and 33% among all children studied. Though all children with anaemia in this study could be deemed to be at risk of its complications, those with moderate anaemia are particularly at risk of long‐term complications, including death [25]. Thus, the observed high prevalence portends an inherent risk for the population. Although only 3.2% of our study population had severe anaemia, the risk of death is even more alarming for this group. These observations underscore the need for urgent action to curb the prevalence of moderate‐to‐severe anaemia and thereby mitigate these risks.

### 4.2. Health, Demographics and Anaemia

The association between health and demographic factors and anaemia in children under five is well established. Specific factors, however, vary by locality and region. Our study identified that an increase of 1 standard deviation in age was associated with a significant reduction of 0.19 in the odds of severe anaemia in this cohort. The association between younger age and increasing severity of anaemia is well known and has been reported in many studies worldwide [26, 27]. Though age‐related anaemia may be considered a quasi‐nonmodifiable risk, various treatment strategies have been successfully implemented to reduce it. These include iron, vitamin C, folic acid supplementation [28], regular monitoring and dietary diversification. Various studies have shown that the burden of anaemia is reduced dramatically with iron and trace element supplementation [29]. Indeed, the low incidence of helminthiasis and the absence of schistosomiasis in this rural community may primarily be due to sustained mass deworming practices in Ghana. This calls for a concerted effort among stakeholders to promulgate and sustain such policies.

Our study found two nutrition‐related factors associated with anaemia. First, the children who engage in communal eating rather than eating from their own bowls. Although not widely studied, this effect may be applicable in some Ghanaian settings. Communal eating, often done by siblings and close relatives, is common, especially among Ghanaian rural folks [30]. Unfortunately, this practice frequently leads to younger children, who usually require longer eating times and more attention when eating, having inadequate dietary intake of the four main food types and the trace elements necessary for haemoglobin formation. Incorporating this recommendation in educational messages at antenatal and well‐baby clinics may reduce this practice and the incidence of anaemia. The second factor significantly associated with anaemia in our study is stunting in the child. This is a well‐known and studied effect, often attributed to concurrent chronic poor dietary habits, chronic illness and other factors.

Although religion is not universally linked to anaemia, we found that both parents’ religion and maternal religion were separately and roughly associated with anaemia. Children of mothers with the Islamic faith were significantly associated with anaemia. The indirect but significant interplay between dietary practices, healthcare utilisation, sociocultural, economic and maternal education, empowerment, and religious belief lays the foundation for an indirect effect of parental religion and anaemia in the children. In India, the association between maternal autonomy and child haemoglobin levels varied by religion. For example, higher haemoglobin levels were observed among children of Hindu mothers with greater autonomy, and a statistically significant association was found for Muslim mothers in a specific survey year [31]. To our knowledge, the link between religion and anaemia in Ghana has not been previously documented. Therefore, further studies are required to establish whether this has a direct or indirect effect.

The final observed significant predictor of anaemia in this study was the presence of malaria parasitaemia. It is alarming that in our study population, 52.5% of the study participants had malaria parasitaemia. As many as 66.4% had at least one episode of fever within three months before the study. Given that only 32.5% of them visited a health facility during the same period, it can be inferred that symptomatic malaria is very common among these children, but their treatment may be inadequate. Ghana experiences a high burden of seasonal malaria transmission. The exceptionally high parasitaemia could be attributed to the time of conducting this study. May to November is the high malaria transmission season in most of Ghana [32]. This parasitaemia contributes significantly to malaria morbidity and its related chronic and acute anaemia. Some studies have been conducted and have shown promising results in reducing this burden in children under five years [33, 34]. Currently, Ghana does not implement intermittent malaria treatment as a policy for children under five years. Instituting this may be a good way to reduce the high burden of malaria and anaemia.

The high anaemia prevalence in this study underscores a persistent public health issue among preschool children in rural Ghana, despite existing nutritional and malaria efforts. The link between malaria parasitaemia and anaemia highlights the need for ongoing vector control and chemoprevention, especially during peak transmission times. Strong ties between stunting, communal eating and anaemia suggest nutrition education should promote child feeding autonomy and balanced diets, not just food access. The connection between maternal religion and childhood anaemia emphasises cultural and behavioural influences on nutrition, underscoring the need for culturally sensitive community strategies.

Taken together, these findings indicate that integrated programmes combining malaria control, child feeding education, and maternal nutrition counselling could be more effective in reducing anaemia in similar rural settings. Strengthening community‐based growth monitoring and anaemia screening, alongside the provision of iron and micronutrient supplementation, could also help identify and manage at‐risk children early.

Our study extends existing literature by demonstrating that anaemia in this rural Ghanaian setting is driven not only by malaria and nutritional status but also by modifiable feeding behaviours and maternal sociodemographic characteristics, highlighting targets for locally tailored interventions.

The strength of this study lies in its multistage, community‐based design, which involved four communities with different social and economic profiles.

This study has some limitations that should be considered when interpreting the findings. First, the cross‐sectional design restricts the ability to establish causal relationships between the identified predictors and anaemia. Second, some variables, such as recent illness and dietary practices, were based on self‐reported information and might be subject to recall bias. Third, the study was conducted in a single subdistrict, which may limit the generalisability of the findings to other settings. Despite these limitations, the study offers valuable insights into the prevalence and predictors of anaemia among children in the study area.

## 5. Conclusion

Our study determined the prevalence of anaemia and its predictors in children under five years in the Ejura‐Sekyedumase Municipality. We found a very high burden of anaemia in children within the municipality. The independent predictors of anaemia included stunting, communal eating rather than eating from a child’s bowl, younger age, the mother’s religion, and malaria parasitaemia.

The high rates of anaemia and malaria among our study population emphasise the urgent need for comprehensive health strategies. Malaria control measures, such as insecticide‐treated nets, early diagnosis and treatment, and nutritional support, are essential. Primary health facilities could incorporate routine anaemia screening and growth monitoring into child welfare services. The identified predictors can help guide targeted actions, such as promoting proper feeding, enhancing nutrition to prevent stunting, and delivering culturally tailored health education for mothers, particularly those with limited schooling. These efforts highlight the importance of local authorities and policymakers basing decisions on evidence to achieve lasting improvements in child health.

**Nomenclature**
CHPS: Community‐based Health Planning and Services
EDTA: Ethylenediaminetetraacetic acid
RDT: Rapid diagnostic test

## Author Contributions

Eugenia Sly‐Moore was involved in formulating the study idea, planning and executing data collection, and writing the final manuscript. Samuel Blay Nguah was involved in formulating the study idea, planning data collection and analysis and authoring. Haruna Mahama was involved in formulating the study idea, planning data collection and authoring. Sampson Antwi was involved in formulating the study idea, planning data collection and writing the final manuscript.

## Funding

No funding was received for this research.

## Disclosure

All authors approved the final manuscript.

## Ethics Statement

The institutional review board of the Kwame Nkrumah University of Science and Technology (IRB approval no. FWA00002761) gave ethical approval for the study. Also, permissions were sought from the Ejura‐Sekyedumase Municipal and Ashanti Regional Health Directorates. The management of St Luke Hospital, Kasei, and the community leaders were also informed. To be recruited, consenting was done for each child–parent pair, and informed consent was obtained from the legal guardian before recruitment.

## Consent

Please see the Ethics Statement.

## Conflicts of Interest

The authors declare no conflicts of interest.

## Data Availability

The data that support the findings of this study are available on request from the corresponding author. The data are not publicly available due to privacy or ethical restrictions.
